# Genetic dissection of fruit maturity date in apricot (*P. armeniaca* L.) through a Single Primer Enrichment Technology (SPET) approach

**DOI:** 10.1186/s12864-022-08901-1

**Published:** 2022-10-19

**Authors:** Irina Baccichet, Remo Chiozzotto, Davide Scaglione, Daniele Bassi, Laura Rossini, Marco Cirilli

**Affiliations:** 1 Università degli Studi di Milan – DiSAA, Milano, Italy; 2 IGA Technology Services Srl, Udine, Italy

**Keywords:** Apricot, Fruit, Fruit crops genomics, GWAS, Maturity date, SPET

## Abstract

**Background:**

Single primer enrichment technology (SPET) is an emerging and increasingly popular solution for high-throughput targeted genotyping in plants. Although SPET requires *a priori* identification of polymorphisms for probe design, this technology has potentially higher reproducibility and transferability compared to other reduced representation sequencing (RRS) approaches, also enabling the discovery of closely linked polymorphisms surrounding the target one.

**Results:**

The potential for SPET application in fruit trees was evaluated by developing a 25K target SNPs assay to genotype a panel of apricot accessions and progenies. A total of 32,492 polymorphic sites were genotyped in 128 accessions (including 8,188 accessory non-target SNPs) with extremely low levels of missing data and a significant correlation of allelic frequencies compared to whole-genome sequencing data used for array design. Assay performance was further validated by estimating genotyping errors in two biparental progenies, resulting in an overall 1.8% rate. SPET genotyping data were used to infer population structure and to dissect the architecture of fruit maturity date (MD), a quantitative reproductive phenological trait of great agronomical interest in apricot species. Depending on the year, GWAS revealed loci associated to MD on several chromosomes. The QTLs on chromosomes 1 and 4 (the latter explaining most of the phenotypic variability in the panel) were the most consistent over years and were further confirmed by linkage mapping in two segregating progenies.

**Conclusions:**

Besides the utility for marker assisted selection and for paving the way to in-depth studies to clarify the molecular bases of MD trait variation in apricot, the results provide an overview of the performance and reliability of SPET for fruit tree genetics.

**Supplementary Information:**

The online version contains supplementary material available at 10.1186/s12864-022-08901-1.

## Background

The tremendous advance in DNA technologies and the advent of next generation sequencing (NGS) platforms are making possible a comprehensive cataloguing of genetic variation, providing an unprecedented resolution for analyzing ancestry, evolution and trait diversity [1]. Over the past decades, several high-throughput methods have been described for genotyping, being whole-genome re-sequencing (WGRS)—e.g. short-reads sequencing on the Illumina platform and reads alignment to a reference assembly—the most straightforward in species with an available reference genome [2]. Nevertheless, the financial burden associated with amassing sufficient sequence data is still an important drawback, particularly in minor crops. Several techniques have been developed, mostly oriented to the genotyping of single nucleotide polymorphisms (SNPs), the most abundant type of sequence variation in plant genomes [3]. Array-based SNP genotyping platforms have become popular in many fruit crops, having several benefits from rapid high-density genome scans, high call rate and robust allele calling to cost-effectiveness per data point (particularly for large numbers of SNPs and samples) [4]. However, arrays are non-flexible and the costs of chip development (or upgrade) are almost only affordable by (relatively) large consortia, as in the case of peach [5], apple [6], pear [7] or grape [8]. The reduction of genome complexity to increase read depth in certain genomic regions is the basic principle of the reduced representation sequencing (RRS) approaches [9], such as RAD-seq [10], genotyping-by-sequencing (GBS) [11], double-digest RAD sequencing (ddRAD) [12] or SLAF-seq [13]. The common feature of all these methods is genomic complexity reduction by digestion with restriction enzymes and subsequent short-read sequencing of fragments bordering restriction sites [14]. RRS approaches have been widely used in many plant species for genetic studies, such as linkage and association mapping, diversity and population structure and core collection development [15]. Compared to SNP arrays, RRS genotyping has the advantage of not requiring prior knowledge to develop assays and of minor ascertainment bias, although they have also several cons, as reviewed by Lowry et al. [16]. Single primer enrichment technology (SPET, United States Patent 9,650,628), is a relatively recent technology offering a customizable and cost-effective solution for targeted sequencing. This technology requires a priori identification of polymorphisms for probe design. Probes are short DNA sequences of around 40-bases long designed in the regions adjacent to the target variant enabling also the detection of any additional polymorphisms (accessory non-target variants) surrounding the target one. Application of SPET has been increasingly reported in plants, including maize, poplar, oil palm, eggplant and tomato [17–19], either for genotyping germplasm resources (also including wild relatives) or cross-progenies. The customization and scalable probe design can maximize the number of target locations and the sequencing of all SNPs in the genomic regions for which probes have been designed. Moreover, once developed and validated, SPET has potentially higher reproducibility and transferability compared to the other RRS genotyping approaches.

Apricot (*Prunus armeniaca* L.) is an economically relatively important *Prunus* species, with a worldwide production of 4.4 million tons in 2018 [20]. In the last two decades, the apricot varietal landscape has been progressively revolutionized by the introduction of several novel accessions from various breeding programmes [21–24]. Selection for improved productivity (adaptability and floral self-compatibility), *Plum Pox Virus* (etiological agent of Sharka disease) resistance and fruit quality (internal and external) have been the major emphasis of breeders [25], leading to a renewed interest for both the offer of novel fruit types and the extension of the ripening calendar. *Ex situ* apricot collections largely represent the source of parental material for crossing. Several studies have investigated genetic diversity and population structure in various germplasms backgrounds by simple-sequence repeats (SSRs) markers [26–29] and, more recently, RRS [30] or WGRS techniques [31]. All these approaches have allowed to elucidate the domestication history and relationships among the different eco-geographical groups, providing a theoretical basis for an effective use of plant resources. Also, the recent release of high-quality reference genome sequences of some apricot cultivars and other *Armeniaca* species (*P. sibirica* and *P. mandshurica*) [31, 32] has been a milestone for in-depth evolutionary and genomics studies, and the way to associate allelic variation with phenotypic traits. A major challenge for apricot breeding is the development of molecular approaches to assist selection; currently, major efforts have been made for shedding light on the genetic basis of PPV resistance [33–35] and floral self-compatibility [36, 37], leading to the creation of several linkage maps, the identification of major loci and consequent design of marker-assisted selection (MAS) [38, 39], or more advanced genomic selection-based prediction approaches [40]. However, knowledge about genomic regions underlying polygenic traits, such as those that define fruit quality, tree phenology and adaptability to the environment, is still scarce.

In this work, we described the first application of SPET technology in apricot. A collection of accessions and breeding selections was genotyped using 25K probes assessing SPET reliability for analyzing genetic variation and population structure. Furthermore, as a proof-of-concept, genome-wide association studies (GWAS) and Quantitative Trait Locus (QTL) mapping were performed to dissect the genetic architecture of maturity date in a panel of accessions and breeding selections, and two additional segregating progenies.

## Results

### SPET array results

A total of 25K SPET probes were designed, mostly located in gene-rich chromosome regions: SNP type and position based on the apricot genome reference assembly of ‘Chuanzhihong’ cultivar [32] is reported in Supplemental File 1.

**Fig. 1:**
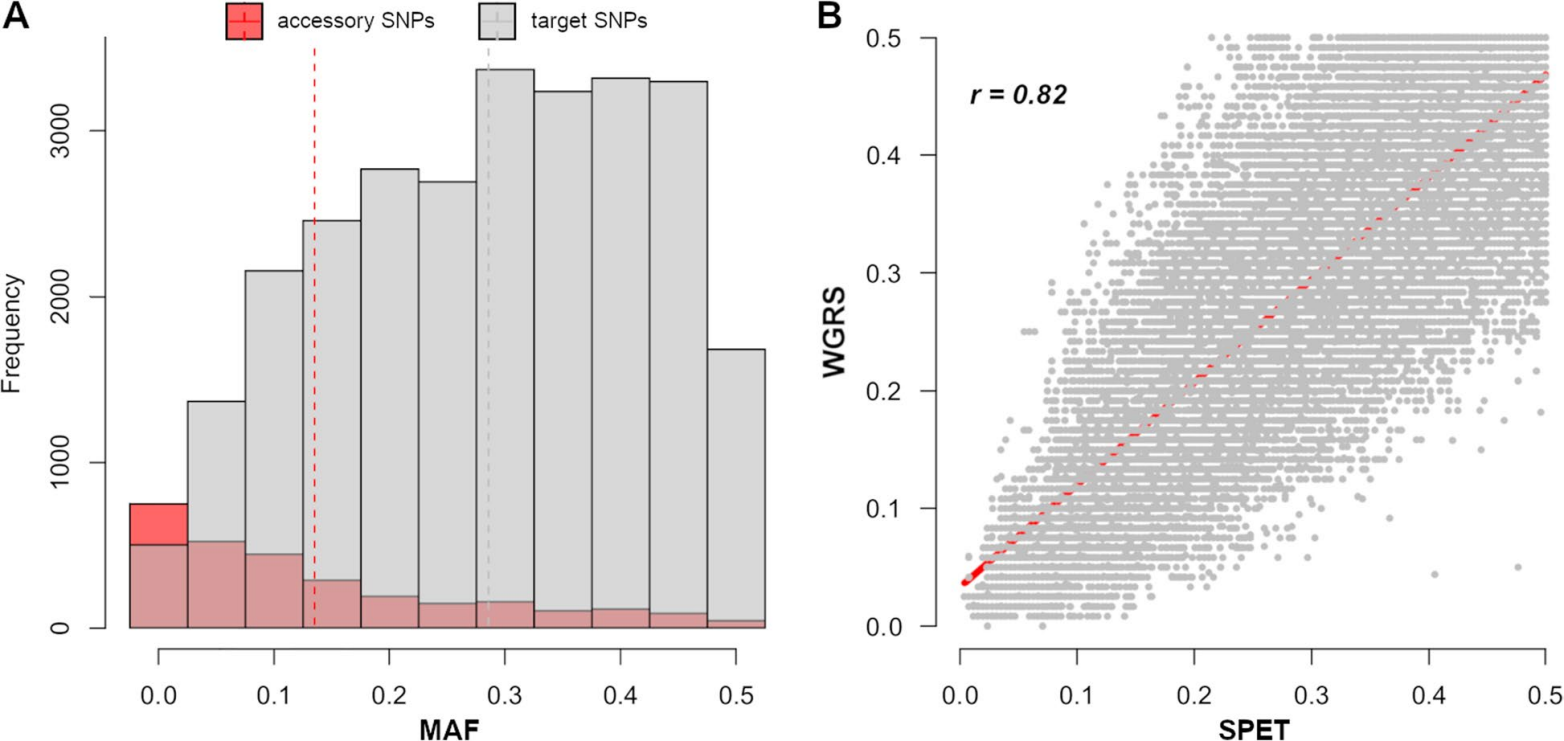
**A** Distribution of minor allele frequencies (MAF) between target (24,304) and accessory (8,188) SNPs in a collection of 128 apricot accessions (grey and pink histograms, respectively). **B** Pearson’s correlation of MAF spectrum at the 32,492 polymorphic sites between the accessions panel (SPET) and the assembled whole-genome re-sequencing (WGRS) data of 66 accessions used for probe design

SPET probes were used to genotype a panel of 128 accessions. After quality filtering and trimming, a total of 206 million Paired End (PE) reads were obtained (an average of 1.6 million reads per sample) with an average mapping rate of 97.9% and a mean depth per site of about 80× (Supplemental File 1). The full set of sequences provides an overall mean 0.6× coverage of the assembled apricot genome (size of 221.9 Mbp). By applying stringent criteria for variant calling and after excluding INDELs, a total of 32,492 polymorphic sites were identified: 24,304 target SNPs plus 8,188 accessory non-targets SNPs. Missingness per site showed extremely low levels, less than 0.01%. The distribution of minor allele frequency (MAF) tended to be similar among the various classes, although sites with MAF < 0.1 were more abundant in accessory SNPs (Fig. 1). Also, MAF spectrum was homogenous across the 8 chromosomes, with an average value of about 0.30, although a certain variability was observed in some regions of chromosomes 2 and 3 (Supplemental Fig. 1). As an estimate of ascertainment bias, the entire allele frequency spectra (in the range 0 – 0.5) in the collection panel were correlated to the original WGRS dataset used for SPET design. Although most of accessory sites were rare SNPs with very low MAF (mostly between 0.01 – 0.02), a significantly strong Pearson’s correlation (*r* = 0.82) was found between SPET and WGRS (Fig. 1).

As validation of the SPET assay reproducibility, a total of 152 seedlings from two segregating progenies were genotyped: 90 seedlings from ‘Fiamma’ (F) × ‘BO 93623033’ (B93) F_1_ cross (hereafter F × B) and 62 seedlings from ‘Lito’ (L) × ‘BO81604311’ (B81) F_1_ cross (L × B). A total of 336 million PE reads were obtained, with an average of 2.2 million per sample (Supplemental File 1). Both the average mapping rate and the mean depth per site were similar to those obtained for the collection panel showing 98.9 and about 90× coverage respectively (Supplemental File 1), with a negligible proportion of missing data (below 0.01%). An approximate estimate of genotyping errors rate was obtained by the evaluation of allelic frequency and Mendelian inconsistency both in segregating and monomorphic SNPs, resulting in an overall 1.8% rate over the 32,492 loci (largely due to singleton).

**Fig. 2:**
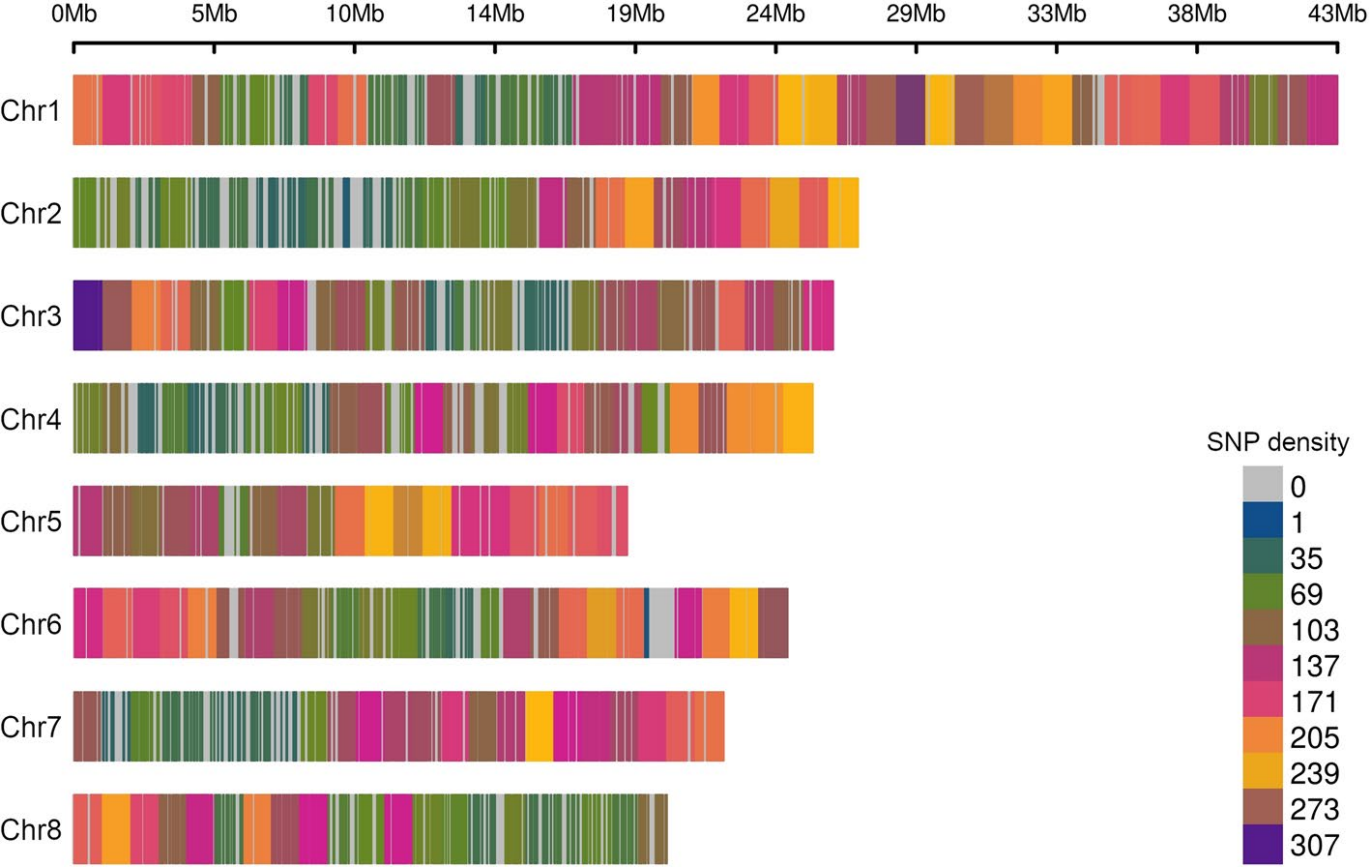
Density of the 25,704 SNPs from 128 accessions across the 8 pseudo-chromosomes in the apricot reference genome sequence [32], reassigned based on the linkage group of *Prunus* reference map [41]

Once filtered for MAF > 0.05, a total of 25,704 clean SNPs were available for genetic analyses (23,556 and 2,148, respectively for target and accessory SNPs). As shown in Fig. 2, SNPs efficiently covered the eight pseudo-chromosomes in the apricot reference genome (recalibrated based on the linkage group of *Prunus* reference map [41]) with an average distance between adjacent markers of 10.6 Kb (ranging from a minimum of 1 to a maximum of 933 Kb). Specifically, 3,885 and 21,819 SNPs were located respectively in intergenic and genic regions, of which 9,415 in exons, 1,760 in UTRs and the remaining in the introns. Linkage disequilibrium (LD) decay was in the order of about 10 Kb for an *R* < 0.2, while the heterozygosity level ranged from 0.12 in the F_3_ self-crossed selection ‘BO06603111’ to 0.51 of ‘Hellin’, with the majority of SNPs showing an heterozygosity level in the range 0.4 – 0.5 (Supplemental Fig.  2).

### Population structure

**Fig. 3:**
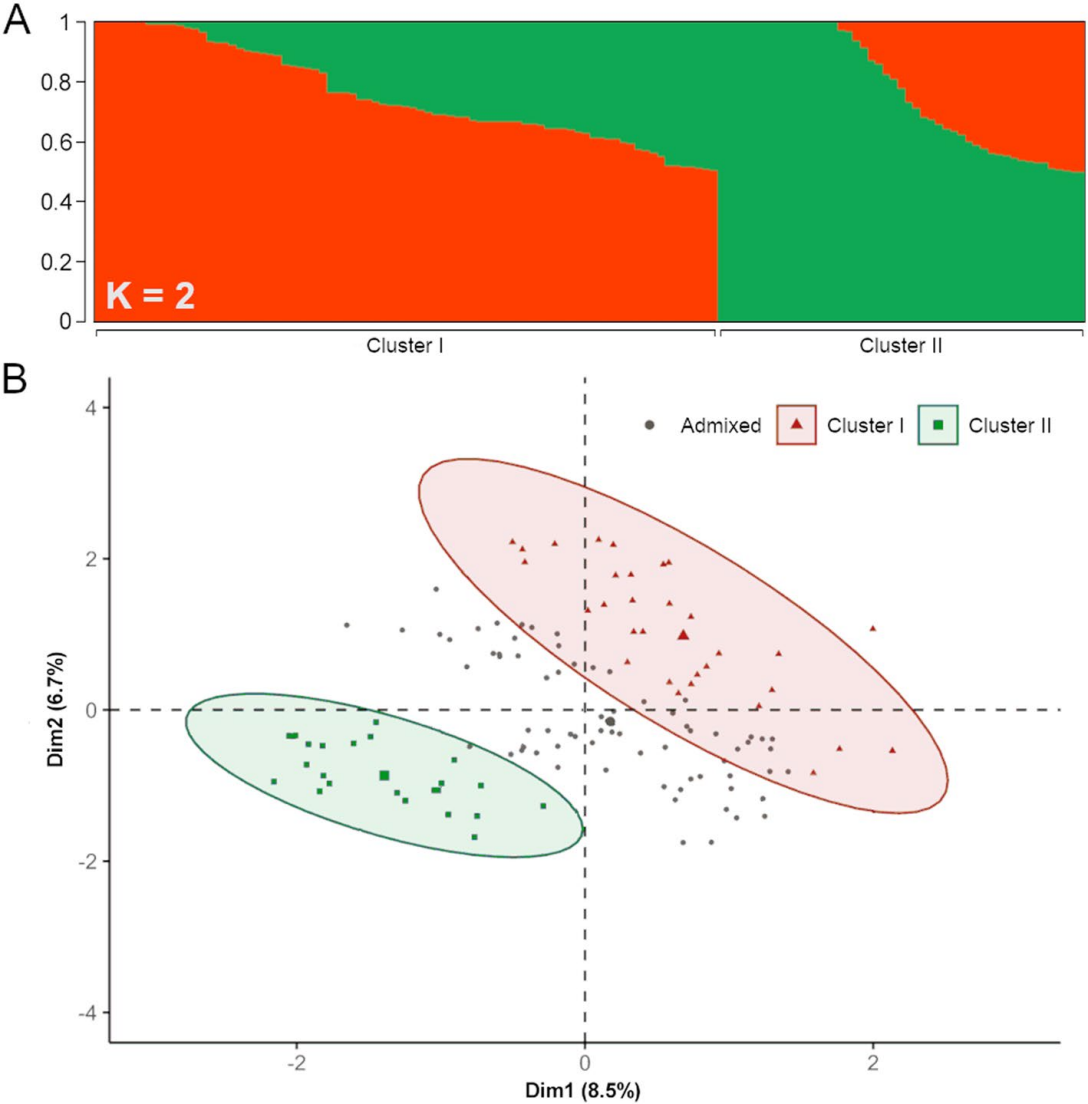
Genetic structure of the apricot accessions panel. **A** Population structure estimated for K (number of a priori cluster) equal to 2 (A) and 4 (**B**). Scatter plot of the first two principal components (Dim1 and Dim2) of the genetic relationship matrix. Red and green colors indicate the two subpopulations (cluster I and II), respectively. Parenthesis report the explained variance

For structure evaluation, the set of 25,704 SNPs were LD-pruned for *R* > 0.3, retaining a total of 1,593 SNPs (Supplemental File 1). Analysis revealed the presence of two to four genetic clusters, as supported by cross-validation procedure for maximizing the predictive accuracy. For a number of a priori cluster (K) equal to 2 and a membership coefficient higher than 0.75 (Supplemental Fig. 3), the panel stratification largely reflects the geographical and/or breeding origin of plant materials, being the first cluster (I) prevalently made up of accessions from Mediterranean and Continental Europe and the second cluster (II) of the individuals derived from North America breeding programs under the effort of introgressing PPV resistance (Fig. 3).

The admixed cluster mostly includes recently released PPV-resistant cultivars and related breeding selections. This pattern of stratification is also captured by the principal component analysis (PCA), with *Dim1* and *Dim2* component capturing 8.5 and 6.7% of the total explained variance (Fig. 3). The clusters I and II were clearly differentiated while admixed individuals occupied a centric position. Stratification patterns were consistent with UPGMA hierarchical clustering, supporting the dendrogram morphology (Supplemental Fig. 4).

### Association mapping of maturity date in apricot

**Fig. 4:**
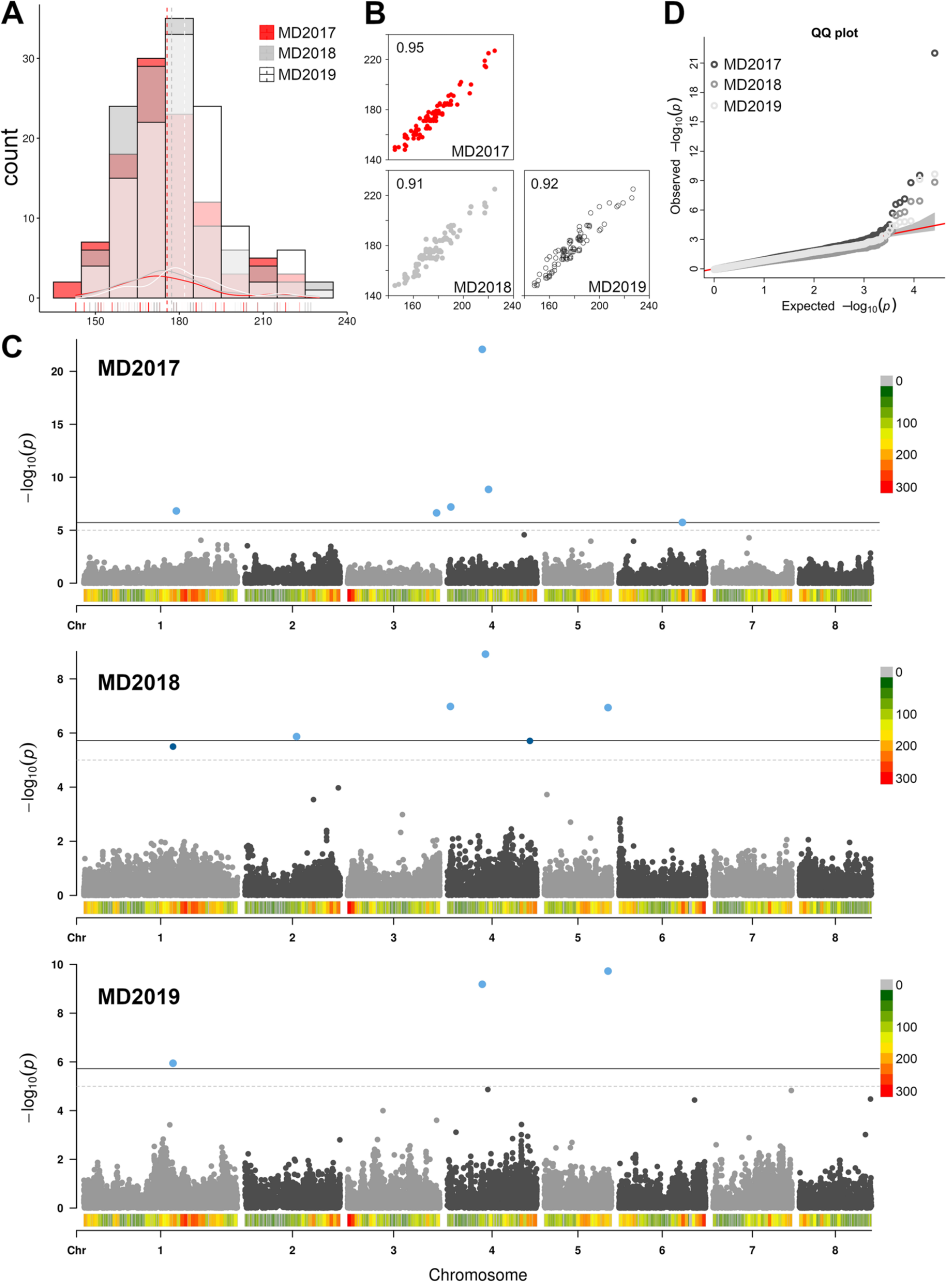
Frequency distribution (**A**) and pairwise Pearson’s correlation coefficients (**B**) of the apricot maturity date (MD) trait in the association mapping panel across the three years of evaluation (2017, 2018 and 2019). Manhattan (**C**) and QQ-plot (**D**) of -log10 *p-values* estimated from the three-year data using FarmCPU algorithm adjusted for population structure. Horizontal lines indicate the Bonferroni’s-adjusted threshold (continuous line, 1.93E^−06^) and permutation test (dashed line, 2.63E^−05^)

Maturity date (MD), expressed in Julian Days (JD) at harvest, was highly correlated among the three years of observation (r-squared higher than 0.9), although mean MD in 2019 was about one week later compared to 2017 and 2018 (Fig. 4). MD range varied between 143—223, 148—227 and 153—230 JD, respectively in 2017, 2018 and 2019 seasons. Across seasons, ‘Pricia’ and ‘Tsunami’ were consistently the earliest ripening cultivars while ‘Augusta 2’ was the latest one. By categorizing MD data into bins of 10 day intervals, distribution was almost normal with a frequency peak around 180 JD (around the II decade of June). Association analyses for MD trait were performed by FarmCPU algorithm using yearly records and the overall validity evaluated through quantile–quantile (QQ) plot inspection. Significant hits above the Bonferroni or FDR significance thresholds (1.91E^−06^ and 2.05E^−05^) were detected on chromosomes (chr) 1, 2, 3, 4, 5 and 7, depending on the year (Fig. 4 and Table 1).

**Table 1:** Association statistics of loci most significantly associated with the MD trait in the accession panel

SNP id	Type	Chr	Position	MAF	*p-value*	gene model
**MD2017**						
SNP25197	C/A	4	9,819,302	0.20	8.35E^−23^	*PARG10387*
SNP25581	C/T	4	11,574,290	0.46	1.40E^−09^	*PARG10602*
SNP23840	T/C	4	1,037,869	0.39	6.38E^−08^	*PARG09400*
SNP16361	C/T	1	25,852,231	0.48	1.51E^−07^	*PARG06739*
SNP37424	G/T	3	24,907,655	0.33	2.32E^−06^	*PARG16093*
SNP5424	G/T	6	17,760,184	0.27	1.83E^−06^	*PARG02232*
**MD2018**						
SNP25460	G/C	4	10,712,890	0.44	1.24E^−09^	*PARG10515*
SNP23816	A/T	4	910,675	0.28	1.04E^−07^	Intergenic
SNP56480	A/G	5	17,821,953	0.22	1.15E^−07^	Intergenic
SNP39805	A/G	2	14,480,994	0.29	1.35E^−06^	*PARG17886*
SNP29182	G/T	4	23,146,124	0.29	1.95E^−06^	*PARG12303*
SNP15833	C/A	1	24,921,422	0.42	3.17E^−06^	Intergenic
**MD2019**						
SNP56480	A/G	5	17,821,953	0.22	1.87E^−10^	Intergenic
SNP25197	C/A	4	9,819,302	0.20	6.50E^−10^	*PARG10387*
SNP15833	C/A	1	24,921,422	0.42	1.13E^−06^	Intergenic

**Fig. 5:**
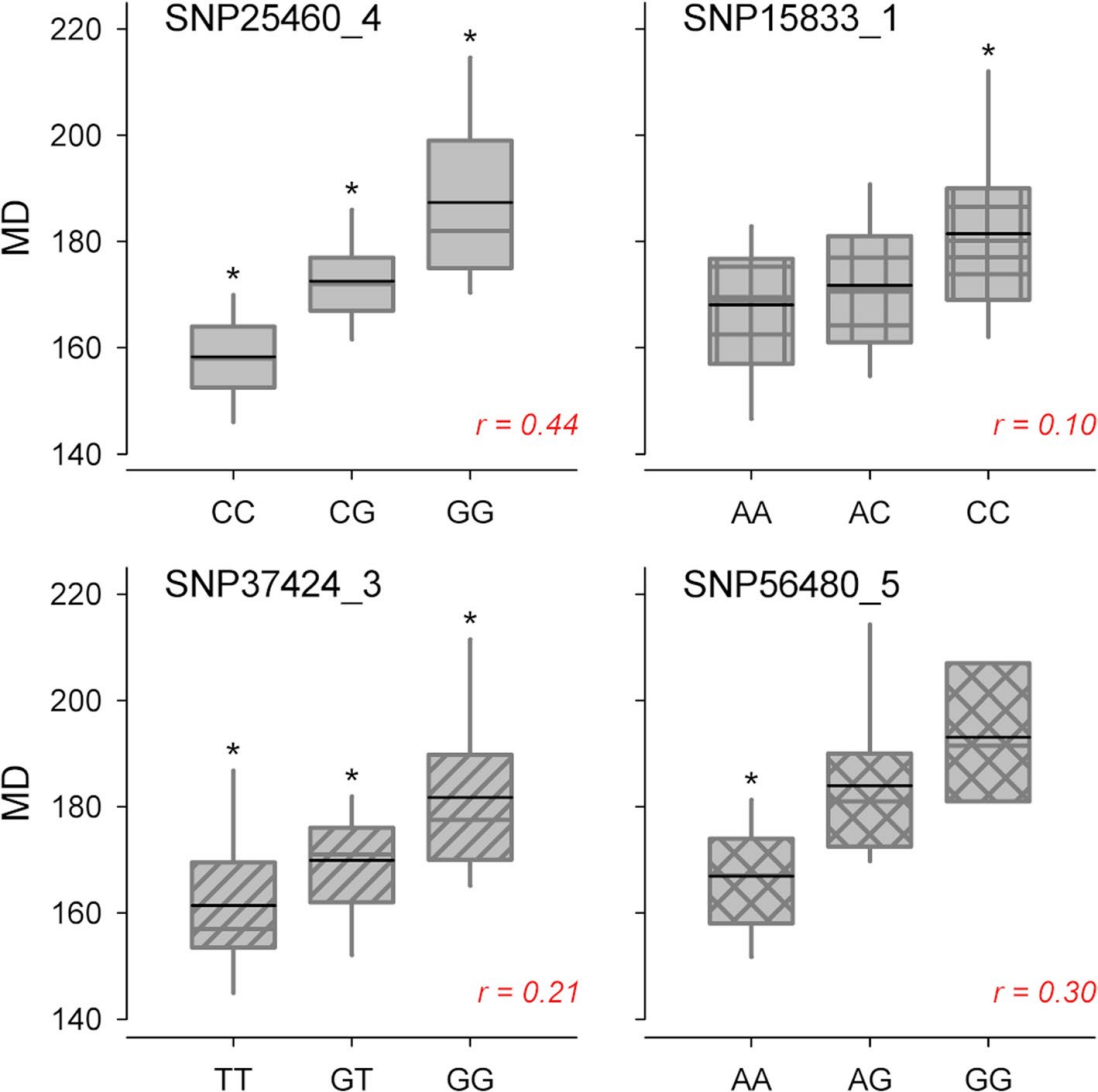
Box-plots of markers-trait association for the four top SNPs detected by GWAS (SNP25460, chr 4; SNP15833, chr 1; SNP37424, chr 3 and SNP56480, chr 5) with across-years averaged maturity date records in the panel of 128 accessions. Asterisks indicate significant differences between segregating classes (*p* < 0.01) as inferred by one-way ANOVA

As estimated by REML analyses, cumulative heritability explained by all SNP loci in each year was 0.82, 0.66 and 0.72, respectively. The signal on chr 1 was consistent across the 3 years of observation, although the top SNP peaks were not identical: SNP16361 (located at 25,852,231 bp) in 2017, SNP15833 (at 24,921,422) in 2018 and 2019. Moreover, a main signal in the middle of chr 4 was stable across years, peaking at SNP25197 (at 9,819,302 bp) in 2017 and 2019, and at SNP25460 (at 10,712,890 bp) in 2018. However, others nearby SNPs beyond the significance threshold were observed at this locus (Table 1). Among the other identified loci, the association on chr 5 at SNP56480 (17,821,953 bp) was detected in two years (2018 and 2019) likewise the one at the proximal end of chr 4, peaking at SNP23840 (1,037,869 bp) and SNP23816 (910,675 bp). LD around top associated SNPs on chr 1 and 4 were also explored to delimit QTL intervals and to identify haplotype blocks, although no clear pattern was observed (Supplemental Fig. 5). For an approximate estimation of SNP effect, a linear regression under additive model was fitted at the most significant SNPs using MD best linear unbiased prediction (BLUP). The model estimated a major effect for SNP25460 locus in the center of chr 4 (r-square of 0.44), followed by SNP56480 on chr 5 (0.30), SNP37424 on chr 3 (0.21) and SNP16361 on chr 1 (0.10) (Fig. 5). Other signals showed significant but lower effects, with an r-square lower than 0.10.

### Linkage mapping for maturity date in two segregating progenies

The SPET array was also used for QTL mapping in the two progenies F×B and L×B, both segregating for MD.

**Fig. 6:**
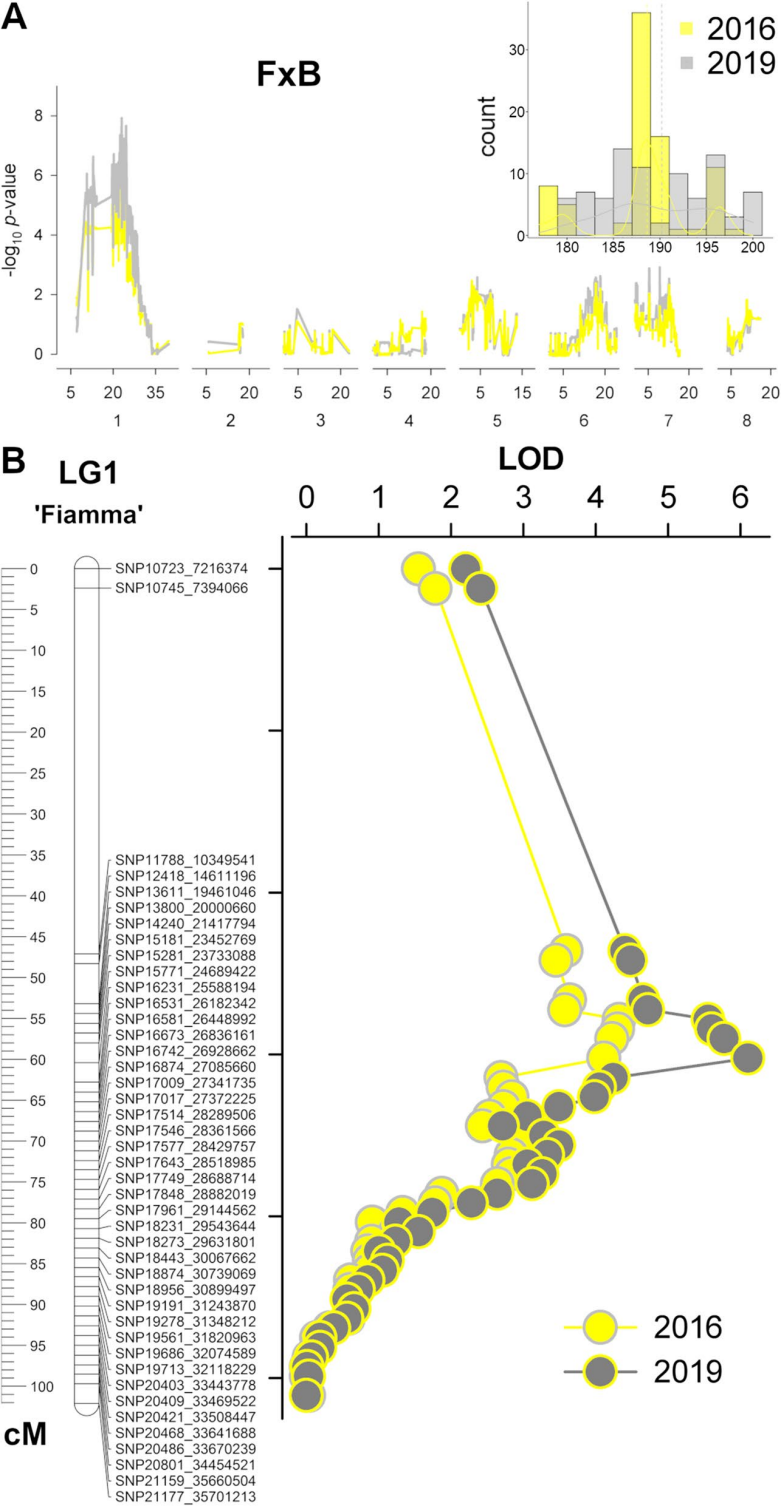
**A** Frequency distribution of maturity date (MD) trait in the ‘Fiamma’ × ‘BO93623033’ (F×B) segregating progenies in the two years of evaluation (right panel). Single-marker analyses of markers-trait association in ‘Fiamma’ parent using 5,157 SPET-derived lm × ll type SNPs markers. **B** Map of linkage group 1 (LG1) and LOD profile of QTL mapping for maturity date (MD) trait (*n* = 90 seedlings)

In F×B, MD spanned a period of about two decades, roughly comprised in the range 180 – 200 JD, being highly correlated in the two analyzed seasons (r-squared 0.89), although distribution in both years was not normal (Fig. 6).

Single marker analysis (SMA) was separately performed in F and B93 to test the association with MD variations. After excluding 15,448 monomorphic and 1,250 markers heterozygous in both parents (i.e. hk × hk according to JoinMap software code), a total of 5,157 (lm × ll) and 4,964 (nn × np) markers were respectively used for F and B93 parents. A SNP cluster on chr 1 was only detected in the F parent, peaking at 23.2 Mb in 2016 and 24.5 in 2019 (*p*-value of 5.8E^−06^ and 7.03E^−07^, respectively) (Fig. 6). A less significant peak on the same position was also detected in B93, although only in 2019. A linkage map of chr 1 (LG1) was built for both F and B93 parents, resulting in a total length of 102.2 and 153 cM, and an average marker density of 2.4 and 3.1 cM. SNP marker order was in good agreement with their physical position in the *P. armeniaca* reference genome, although some gaps larger than 5 Mb of physical distance were identified on the LG1 top (0 – 7 Mb) and bottom (35 – 43 Mb). Interval mapping using the LG1 map highlighted a QTL in the region overlapping the SNP clusters detected with single marker analysis, locating qMD1.1 QTL within the 1.5-LOD interval comprised between 47.1 and 60 cM in 2016 and between 54.4 and 60 cM in 2019 (Fig. 6). SNP peaks at markers SNP15771 (24.6 Mb, 60 cM) and SNP14240 (21.4 Mb, 55.6 cM) showed a percentage of explained phenotypic variance (PEV) of 21.8 and 28.2, respectively. A QTL on the same position was also detected in B93, peaking at SNP15289 (23,870,659, 84.9 cM) although the LOD (2.03) was below the significance threshold. Linkage maps were also built for the other pseudo-chromosomes: maps covered a total of 429 and 468 cM in F and B93 (LG4 not covered), with an overall average marker density of 2.5 and 2.15 cM, respectively (Supplemental Fig. 6).

**Fig. 7:**
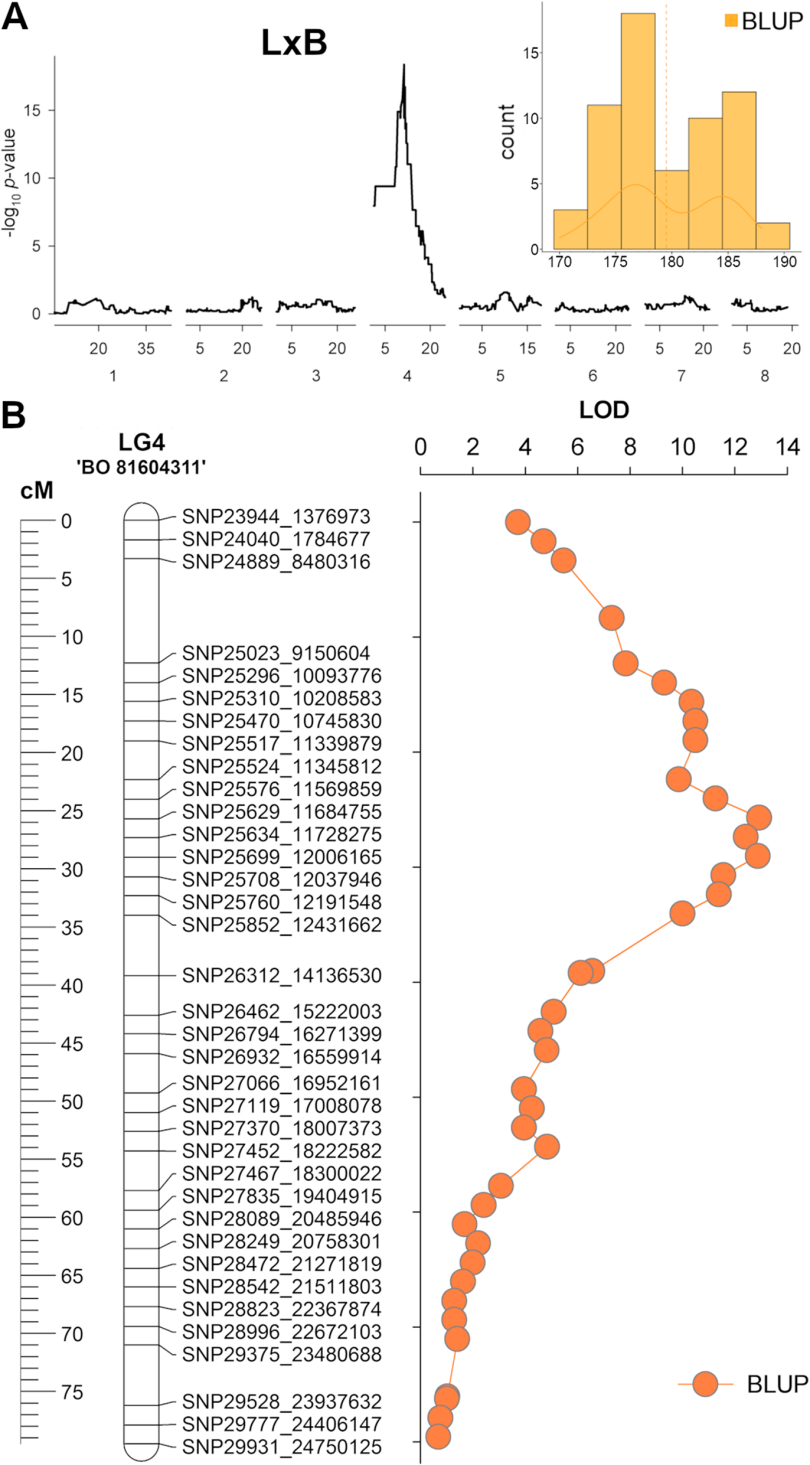
**A** Multiple-year best-linear unbiased prediction (BLUP) of maturity date (MD) trait in the ‘Lito’ × ‘BO81604311’ (L×B) segregating progenies (right panel). Single-marker analyses of markers-trait association in ‘BO 81604311’ parent. **B** Map of linkage group 4 (LG4) constructed from 4,757 SPET-derived nn × np type SNPs markers and LOD profile of QTL mapping for MD trait (*n* = 62 seedlings)

In L×B, best linear unbiased predictor (BLUP) of 5 years MD data showed an almost bimodal distribution (Fig. 7). SMA was separately performed on L and B81 parents using a total of 3,456 (lm × ll) and 4,757 (nn × np) markers. Analyses showed a SNP cluster on chr 4 of B81, peaking at SNP25503 (11.3 Mb, *p*-value of 5.70E^−19^) (Fig. 7) while no evident signals were detected in L parent. A linkage map (LG4) of chr 4 was built for ‘BO81604311’, resulting in a total length of 71.2 cM, with an average marker density of 2.2 cM. Interval mapping using B81 LG4 map located qMD4.1 QTL in the 1.5-LOD interval comprised between markers SNP25629 and SNP25847 (in the physical region 11.6—12.4 Mb) with maximum LOD at marker SNP25704 (12.02 Mb, 29.0 cM) (Fig. 7) and PEV of 62.2%. Linkage maps were also built for the other LG groups: maps covered a total of 586 and 729 cM in L and B81, with an overall average marker density of 2.55 and 2.54 cM, respectively (Supplemental Fig. 7). Even in L×B, marker order on the parental linkage maps largely agreed with the physical position on apricot genome.

## Discussions

### SPET approach for apricot genotyping

In the last decade, the considerable expansion of apricot cultivation has renewed the breeding interest for improving agronomical and pomological traits, as well as the adaptability to different environmental conditions. As in other fruit tree species, the increase of knowledge on the genetic bases of traits variation and the development of molecular markers to assist selection could represent a useful strategy to complement and boost conventional breeding [25, 42]. Also, deploying genomics-assisted breeding approaches and association studies holds a significant promise to unravel complex quantitative traits, identifying favorable haplotypes in promising materials or estimating their breeding values [40]. In spite of the enormous progresses in DNA technologies, high-throughput genotyping approaches have been rarely applied in apricot, where most genetic studies relied on RAPDs, AFLPs, RFLPs or SSRs markers and low-density linkage maps [37, 43–46]. Recently, GBS approaches have been applied for QTL mapping studies and high-density linkage maps development in apricot to generate low-costs SNPs of sufficient quantity and quality for genetic studies [47, 48]. Despite their undoubted advantages (particularly compared to low-density genotyping methods), RRS techniques still have major pitfalls, including: allelic dropout (i.e. polymorphisms in the restriction enzyme recognition site which prevent cutting); PCR duplication and variance in coverage (sometimes with handling of missing data), which can lead to allelic biases; the random distribution of restriction enzyme sites on the genome, and thus the inability to target markers localized within genes, or having a functional significance [16, 49, 50]. In addition, regions covered by GBS markers might not be the same across different studies, making comparisons harder. In consideration of positive experiences in other *Rosaceae*, the development of a SNP-array platform for apricot has been proposed by some research groups, but never realized due to the difficulties of assembling a large consortium in such a minor species. In this work, building upon the recently released high-quality reference genome sequences of some apricot cultivars, a SPET approach was, for the first time, developed and applied for apricot genotyping. While a direct comparison with other approaches is beyond the scope of this work, SPET has the ability of multiplexing samples in a single sequencing run, genotyping tens of thousands of probes with a good coverage, and the advantage of combining targeted SNP analysis and complexity reduction, respectively typical of SNPs array and RRS approaches [17].

The choice of 25K probes for SPET assay was based on the search for a compromise between cost-effectiveness (i.e. total reads number, coverage per site and per sample), prior knowledge on the extent of LD decay in apricot (around 0.1 Kb, [35]), as well as the typical range of panel sizes available for genetics and genomics studies. This probe design allowed to successfully target more than 32K SNPs, of which 21K located in genic regions, including 9,415 within exons. These SNPs may be associated to causative mutations, given their potential to modify protein sequence. Another crucial criterion was a uniform distribution of markers across the genome, in order to facilitate genetic analyses. The average gap size of about 10Kb across all chromosomes provides enough power for association mapping and/or genetic relatedness studies, although a few gaps remain in some regions with low polymorphism levels (with a maximum of 0.9 Mb on chr 6). In addition, very low level of missing data (0.02%) was obtained, greatly facilitating downstream analyses. Untargeted SNPs were also found, confirming the technique ability to discover novel polymorphisms.

Ascertainment bias (i.e. the systematic deviation of population genetic statistics from theoretical expectations) is the main limiting factor of genome complexity reduction approaches, caused by the non-random selection of SNPs or individuals set [51]. However, the allele frequency spectra obtained after SNP discovery in the collection panel was highly similar to the original WGRS spectrum used for SPET array design, indicating a putative limitation of ascertainment bias effects. This could be explained either by the LD-based filtering performed on whole-genome polymorphisms and/or the relatively large dataset of accessions used for probe design (high-coverage WGRS data of 66 genotypes), most of them present within the genetic pools of our collection. Indeed, population structure and phylogenetic relationships were largely congruent with previous literature reports on apricot diversity and breeding history [29, 35, 52]. Nevertheless, the accessions panel did not include wild apricot species or materials of recent introgression and, thus, the reliability of the SPET array for genetic and population genomics studies remains to be evaluated. In case, the flexibility of SPET design will allow further customization of the array, such as the replacement and/or integration of novel polymorphisms from broader genetic backgrounds or relevant QTL-associated variants.

SPET technology was also used for SNP linkage map construction in two segregating progenies. The obtained parental maps showed metrics comparable with those recently obtained through GBS approaches [47, 48] in terms of marker density, map length and gaps presence. Marker order maps also showed high collinearity with the *P. armeniaca* reference genome. Collectively, they provide a sufficiently robust framework for downstream QTL analyses.

### Genetic dissection of maturity date trait in apricot

Maturity date (MD) was dissected as a proof-of-concept of the utility of SPET approach for QTL mapping studies. MD is a relevant trait for fruit marketability, as early ripening cultivars usually command the highest prices while late-maturing cultivars allow the extension of the harvesting season [53]. Understanding the genetic architecture of MD is an important target to assist selection, considering the broad and only partially exploited variability within apricot gene pools. To our knowledge, this is the first attempt to dissect MD trait at germplasm level in apricot, since previous studies have involved bi-parental segregating progenies. GWAS detected stable loci for MD trait on chromosome 1 (qMD1.1) and 4 (qMD4.1) and, depending on the year of observation, additional QTLs in chromosome 2, 3, 4, 5 and 7. Cumulative heritability explained by significant SNP loci was high, ranging from 0.66 in 2018 to 0.82 in 2017. LD pattern in the regions around top associated SNPs did not allow confident delimitation of QTL intervals and reliable inference of haplotypes.

A major effect locus for MD was found in middle of chr 4 (qMD4.1), with associated SNPs distributed in a region of 1.7 Mb from SNP25197 (at 9,819,302) to SNP25581 (at 11,574,290 bp). However, the LD pattern does not allow to exclude the presence of multiple alleles and/or QTLs at this locus. Consistent with GWAS results, a major QTL in this same region segregated in the L×B progeny, where it explained most of the observed PEV for MD (about 62%). A major QTL on linkage group (LG) 4 associated to ripening date was previously reported close to the UDAp439 marker in the cross ‘Goldrich’ × ‘Moniqui’ and also in the same L×B progeny [54]. This major QTL was also mapped in the populations ‘Z701-1’ × ‘Palsteyn’ (located between UDA021 and UDAp439 markers) [55], ‘Bergeron’ × ‘Currot’ (B×C) and in ‘Goldrich’ × ‘Currot’ (G×C) (with markers S4_9061773 and S4_11947345 being the most significant, respectively [56]. Based on our results and these previous works, the QTLs detected on chromosome 4 in our association panel and in different segregating progenies likely correspond to the same locus (qMD4.1). A major QTL in the collinear region of apricot LG4 has been repeatedly reported in other *Prunus* species, such as peach and hybrids [54, 57–59], almond [60] and cherry [61, 62], suggesting a shared mechanism for the control of fruit ripening. In peach, qMD4.1 was fine-mapped to a 220Kb region, identifying a NAC transcription factor allele with an in-frame insertion of 9 bp [63]. At the same locus, a null NAC allele caused by a 26.6Kb deletion co-segregate in heterozygosis with MD [64, 65]. Future efforts will be required to narrow down the qMD4.1 mapping interval in apricot and, possibly, identify the underlying genetic variant(s).

Apart from chr 4, other QTLs have been reported for maturity date in apricot, on LG1, 2, 3, 4, 5, 6 and 7, although affected by the season of observation [54–56]. However, the limited resolution of the linkage maps and the extended confidence interval of QTLs reported in these studies do not allow a direct comparison with GWAS results. In our study, a consistent QTL for MD was found on chr 1 (qMD1.1), although the peak marker SNP16361 (at 25,852,231) explained a low percentage of variance in the panel (about 10%). The presence of qMD1.1 was validated in the F×B progeny, where this QTL accounted for about 20 to 28% of PEV, depending on season. Other two major loci controlling MD architecture were identified on chr 5 (qMD5.1) and 3 (qMD3.1), both explaining over 20% of phenotypic variance in the panel, although they were only detected in one or two or years of data recorded. Additional studies will be required to confirm the presence and effects of these two QTLs, and to infer haplotypes structure at these loci, possibly involving large segregating progenies from different cross-combinations issued from this germplasm and/or a wider GWAS panel enclosing materials from other apricot collections.

## Conclusions

In this work, a SPET targeted genotyping approach was for the first time developed and applied in apricot. The robustness and efficiency of this technique was assessed in a germplasm collection and segregating progenies. Results demonstrate that the SPET approach is a valid alternative to other RRS methods and arrays, highlighting the potential of this technology to provide dense, easy-to-handle genotypic information at the selected polymorphisms and additional untargeted sites. Linkage and association mapping revealed several genomic loci associated to fruit maturity date which may be a starting point for a fine characterization of the genetic basis of trait variation, and ultimately, a useful tool to assist breeding selection and germplasm characterization.

## Material and methods

### Germplasm and phenotypic analyses

The assayed plant material included 128 apricot accessions and two F_1_ apricot progenies from the crosses ‘Fiamma’ × ‘BO93623033’ (F×B) and ‘Lito’ × ‘BO 81604311’ (L×B) (original and extended [66]) with 90 and 62 seedlings, respectively. Accessions derived from MAS.PES apricot germplasm collection located in the experimental farm ‘Brusca’ runned by CRPV (Centro Ricerche Produzioni Vegetali) in Imola (Emilia-Romagna region, Italy). Trees were grafted onto ‘29C Myrobolan’ rootstock, trained according to open vase system, regularly spaced at distance of 4 × 2.5 m (within and between rows, respectively) and managed according to standard cultural practices and yearly pruning. The F_1_ seedlings F×B and L×B were planted at distance of 1 × 4 m (within and between rows, respectively) and trained as slender spindle (one stem with short lateral scaffolds). Maturity date (MD) trait was recorded when 2—3% of the fruits on the tree has reached the full physiological maturity, assessed by sensory and visual inspection, also recording I_AD_ (DA-meter, TR-Turoni, Forlì, Italy) and fruit firmness (puncture test by a digital penetrometer). MD was expressed as the number of days from 1 January (Julian days, JD). MD was evaluated in the accession panel for 3 years (2017, 2018 and 2019) and 2 years (2016 and 2019) in 85 F×B seedlings. In L×B, best linear unbiased prediction (BLUP) was used to pool historical phenotypic data of 58 seedlings from multiple-years records (2006, 2007, 2008, 2017 and 2018).

### Setting-up single primer enrichment technology (SPET)

Apricot whole-genome sequencing (WGS) libraries were retrieved from available NCBI resources, specifically from the previous work of Mariette et al. [35] (PRJNA292050 bioproject) including available Illumina WGS data of 66 accessions; in addition, RNA-seq data from various studies were also included (Supplemental File 1). Data processing was performed as previously described [67]. Briefly, raw reads were quality filtered, trimmed with Trimmomatic and mapped onto the apricot reference ‘Chuanzhihong’ genome V1.0 [32] using the Burrows − Wheeler Aligner (BWA)-MEM algorithm with default parameters. After duplicate removal and indexing of mapped reads with Picard tools, genomic variants were identified by combining the variant-calling algorithms GATK-Haplotype Caller and Freebayes. Polymorphisms were discovered using a joint-calling procedure and quality-filtered according to Best Practice Guidelines, retaining only simple biallelic SNPs. Genomic and transcriptomic data were separately assembled and intersected through a custom Perl script. For SPET assay design, SNP selection criteria were based on: (i) a total number of 25K loci; ii) regularly spaced positions: SNPs within CDS or intergenic regions had to be at least 5 kb and 10 Kb apart from other selected; iii) SNP loci that allowed to design probes on both left and right borders and containing two flanking SNPs; iv) MAF (minor allele frequency) in the range of 0.10—0.90 without an excess of heterozygosity (avoid potential paralogous). Eligible polymorphic sites were forwarded for probe design to NuGen (San Carlos, CA, United States).

### DNA extraction, library construction preparation, SPET sequencing and SNP calling

DNA was extracted from young leaves using the modified CTAB method [68]. Libraries were prepared according to the Ovation Rapid Library Systems (NuGen) specifications. The main steps of streamlined workflow were previously described in Barchi et al. [18]. For the genotyping of the whole set of accessions with the custom 25K probe sets, sequencing was performed at IGA Technology Services (IGATech, Udine, Italy) facilities with Illumina HiSeq 2500 platform (Illumina Inc., San Diego, CA, United States) in single-end mode (150 bp). BCL files from the instruments were processed using the manufacturer’s pipeline software to generate FASTQ sequence files. Base calling and de-multiplexing were carried out using the standard Illumina pipeline previously described, except for SNP calling, obtained using the variant discovery tool ‘UnifiedGenotyper’ in GATK 3.8. After SNP selection using the filter expression (‘QD < 2.0 || MQ < 40.0 || MQRankSum <  − 12.5’), high confidence SNPs were extracted using min-meanDP 30 and max-missing 0.90 and non-ref-ac-any 1. The mean individual coverage at target sites was calculated using *bedtools* v2.26.0 [69].

### Genetic diversity and population structure analyses

Genetic diversity measures on VCF dataset were performed by the Geno Summary tool implemented in Tassel v5.2.15 [70], including the estimation of expected and observed heterozygosity, MAF and missingness per site at all marker loci. Population structure was inferred through ADMIXTURE software v1.22 [71] using 1,593 SNPs obtained from LD pruning (> 0.3). K values from 2 to 6 were inputted to identify the number of a priori genetic clusters that maximizes the predictive accuracy. The phylogenetic tree was built from a pairwise genetic distance matrix between individuals clustered with NJ method in Tassel v5.2.15, with bootstrap replication and tree reconstruction in MEGA7 software [72]. Principal component analysis (PCA) and linkage disequilibrium decay over distance were calculated in GAPIT [73] using the filtered SNPs dataset (25,704). Intra-chromosomal LD patterns were estimated and visualized using HAPLOVIEW [74].

### Genome-wide association study

The fixed and random model Circulating Probability Unification (FarmCPU) algorithm implemented in GAPIT R package, was used for association analyses [73]. Fixed effects from population structure were included as covariates by using the first two PCs from Q-matrix. The observed *vs* expected *p*-values distribution under null hypothesis were compared through quantile–quantile (QQ) plot inspection to evaluate GWAS performance. Thresholds for SNP significance were calculated based on a conservative Bonferroni’s correction for a type I error rate and less stringent permutation test. Manhattan plots were designed using MVP R package [75]. SNP-based broad-sense heritability was estimated by GREML method using GCTA tool (v1.93.2), after fitting top SNPs. Statistical significance of single-marker-trait associations were inferred using one-way ANOVA with post-hoc Tukey’s test.

### Linkage map construction and QTL-mapping

Linkage maps were constructed following the two-way pseudo-testcross strategy for outcrossing species (CP) using JoinMap v4.1 [76]. The input file generated from VCF analysis was manually curated and filtered, retaining only markers polymorphic in one parent (i.e. lm × ll and nn × np configurations, respectively for the seed and pollen parents). Markers were then filtered based on identical loci (> 0.95 similarity threshold) and distorted markers (1:1 ratio, chi-square goodness-of-fit tests at *p *≤ 0.05). Linkage groups were defined using a minimum logarithm of odds (LOD) value of 10.0 using the regression algorithm (Kosambi mapping function), with a recombination frequency threshold of 0.4, LOD value of 1.0 and a goodness-of-fit jump of 5.0. The positions of markers in the F×B and L×B linkage maps were aligned with their position on the apricot reference genome [32]. Single-marker analysis was performed in Tassel v5.2.15 using GLM algorithm. The MapQTL v6.0 software [77] was used for detecting QTLs. Significance thresholds were calculated by random permutation test (PT) with 10,000 replicates considering the genome-wide LOD scores corresponding to *p* = 0.05. The interval mapping (IM) function was employed for QTLs detection with 95% significance and estimation of the percentage of phenotypic variation. MapChart v2.1 software [78] was used to draw the mapped QTLs and the LOD plots.

## Supplementary Information

**Additional file 1.**

## Data Availability

The data that support the findings of this study are available from The University of Milan Institutional Data Access/Ethics Committee. Apricot accessions are cultivated and derived from MAS.PES apricot germplasm collection located in the experimental farm ‘Brusca’ runned by CRPV (Centro Ricerche Produzioni Vegetali) in Imola (Emilia-Romagna region, Italy). The Prof. Daniele Bassi is in charge of the formal management of the plant materials. A voucher specimen of these materials has not been deposited, but available upon reasonable request and with the permission of the University of Milan. The datasets generated and analysed during the current study are available in the Sequence Read Archive (SRA) repository (https://www.ncbi.nlm.nih.gov/sra) under the accession number BioProject ‘PRJNA821640’. The SRA data are publicly available.
