# Supplementary material for: Genetic dissection of fruit maturity date in apricot (P. armeniaca L.) through a Single Primer Enrichment Technology (SPET) approach
Source: BMC Genomics. 2022 Oct 19;23:712. doi: 10.1186/s12864-022-08901-1 (PMC9580121; doi:10.1186/s12864-022-08901-1)
Supplement: Supplementary file 1 — Additional file 1. [file 12864_2022_8901_MOESM1_ESM.zip › SUPPLEMENTAL DATA FILES_Jul_2022.docx]

**SUPPLEMENTAL DATA FILES**

**Supplemental File 1**. Sheet1: SPET assay design, including genomic position, type and annotation of target SNPs based on apricot reference genome assembly (LG^a^, Jiang et al., 2019) and *Prunus* reference genetic map (LG^b^, Dirlewanger et al., 2004). Sheet2: SRA accession number of WGS and transcriptome libraries used for probe design. Sheet3: genetic summary of the unfiltered SNPs dataset (32,492 loci). Sheet4: genetic summary of the MAF-filtered SNPs dataset (25,704 loci). Sheet5: genetic summary of the MAF and LD pruned SNPs dataset (1,593 loci). Sheet6: Genotyping statistics for apricot accessions panel. Sheet7: Genotyping statistics for apricot progenies. Sheet8: Annotation of SNP variants underlining identified GWAS signals.

**Supplemental Figure 1**

**
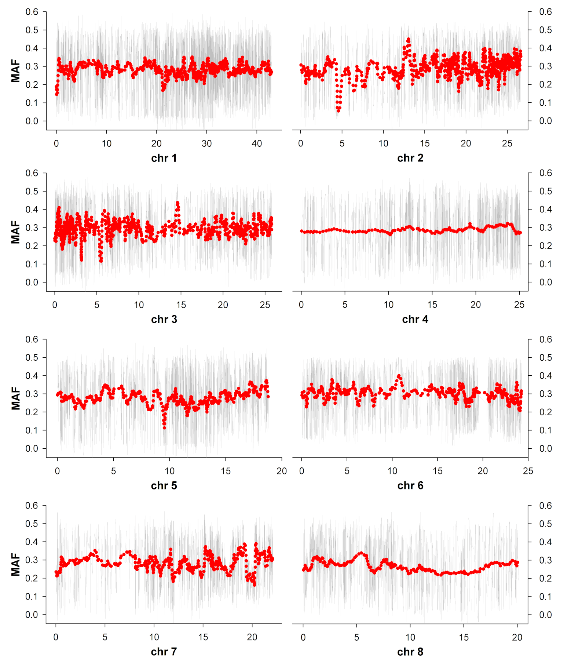
**

**Supplemental Figure 1**. Chromosome-wide distribution of minor allele frequencies (MAF) of the 32,492 SPET-genotyped SNPs in a collection of 128 apricot accessions. MAF was calculated using a moving average with a 50 Kb window.

**Supplemental Figure 2**

**
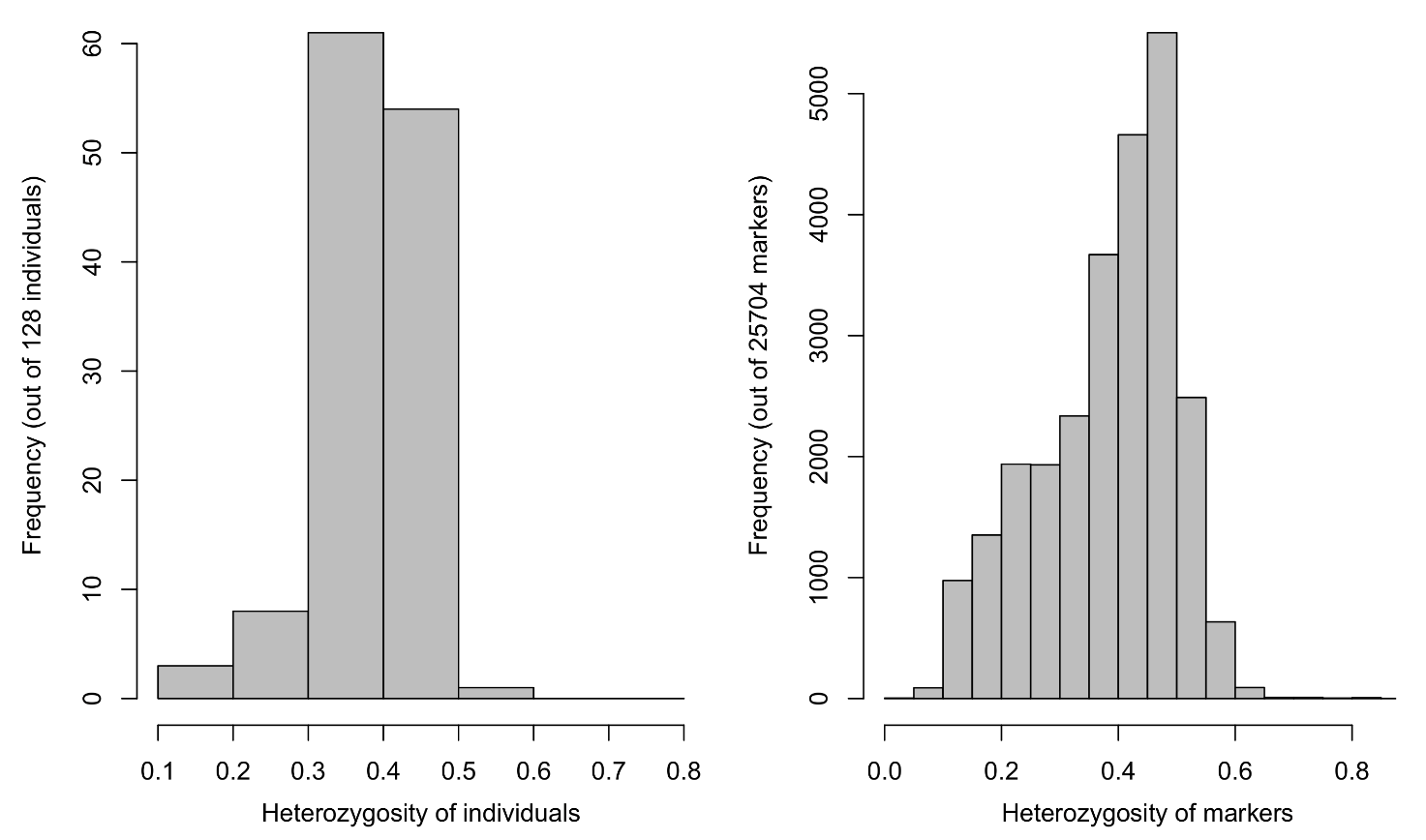
**

**Supplemental Figure 2**. Frequency distribution of observed heterozygosity in the accession panel (left panel) and in the filtered SNPs markers dataset (right panel).

**Supplemental Figure 3**

**
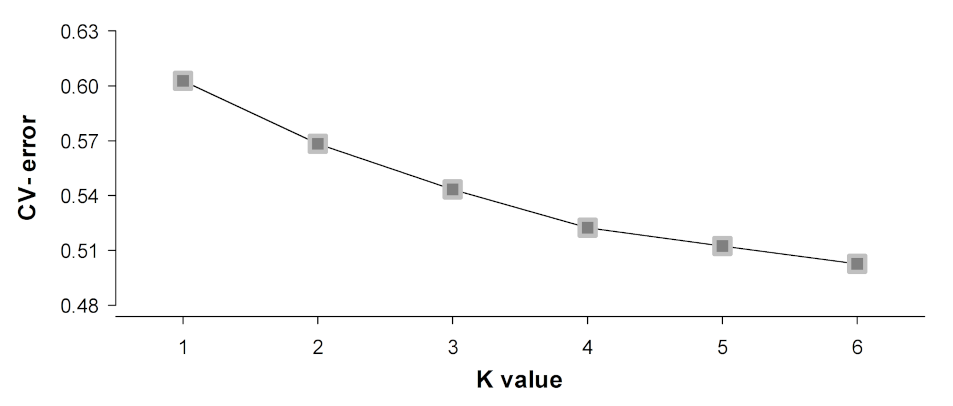
**

**Supplemental Figure 3**. Cross-validation for each *a priori* cluster number (K values) as inferred in ADMIXTURE software v1.22.

**Supplemental Figure 4**

**
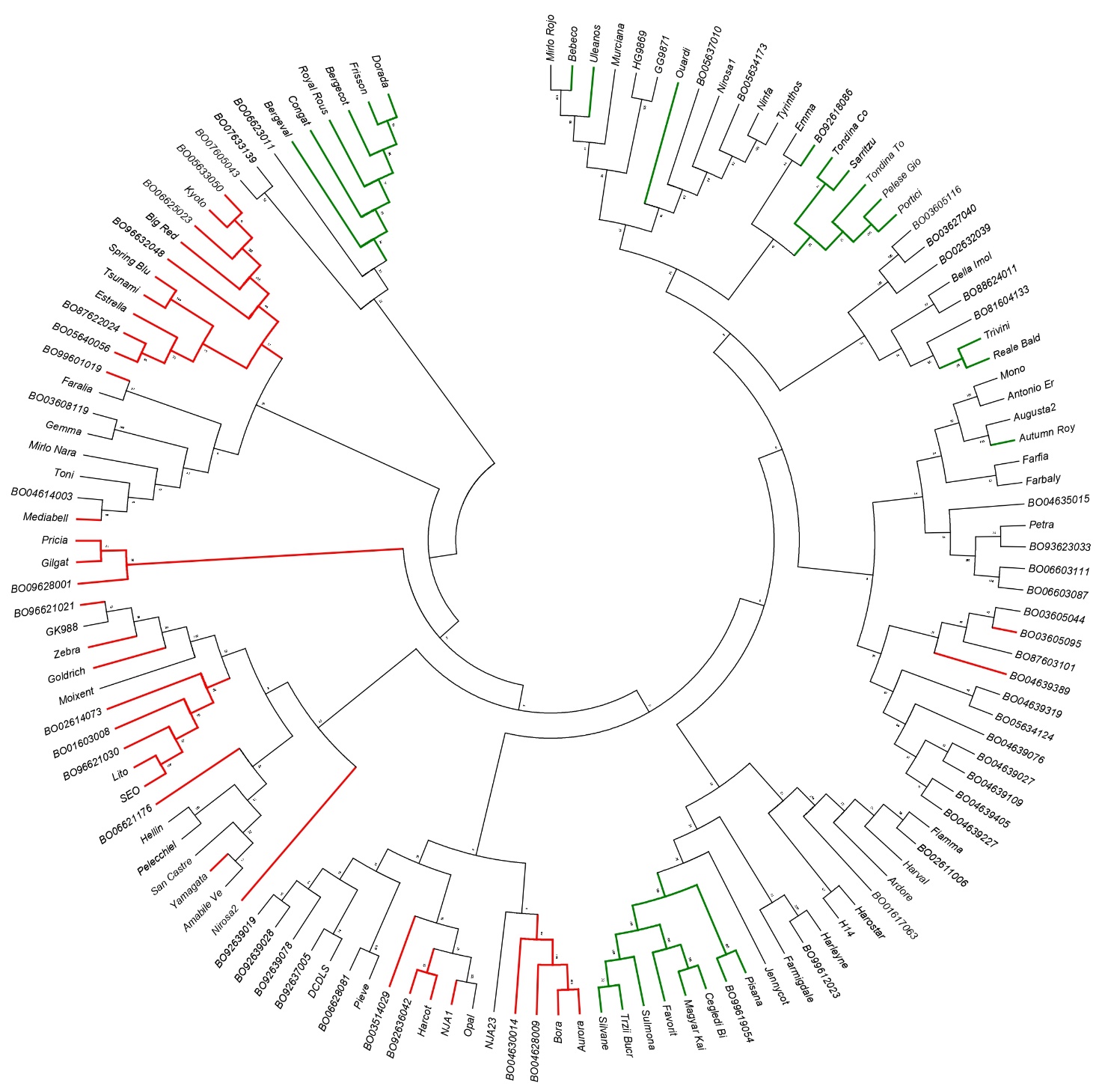
**

**Supplemental Figure 4**. Unrooted Neighbour-Joining (NJ) tree showing the phylogenetic relationship among apricot accessions. Red and green colours mark the assignment of each genotype to the respective cluster I and II.

**Supplemental Figure 5**

**
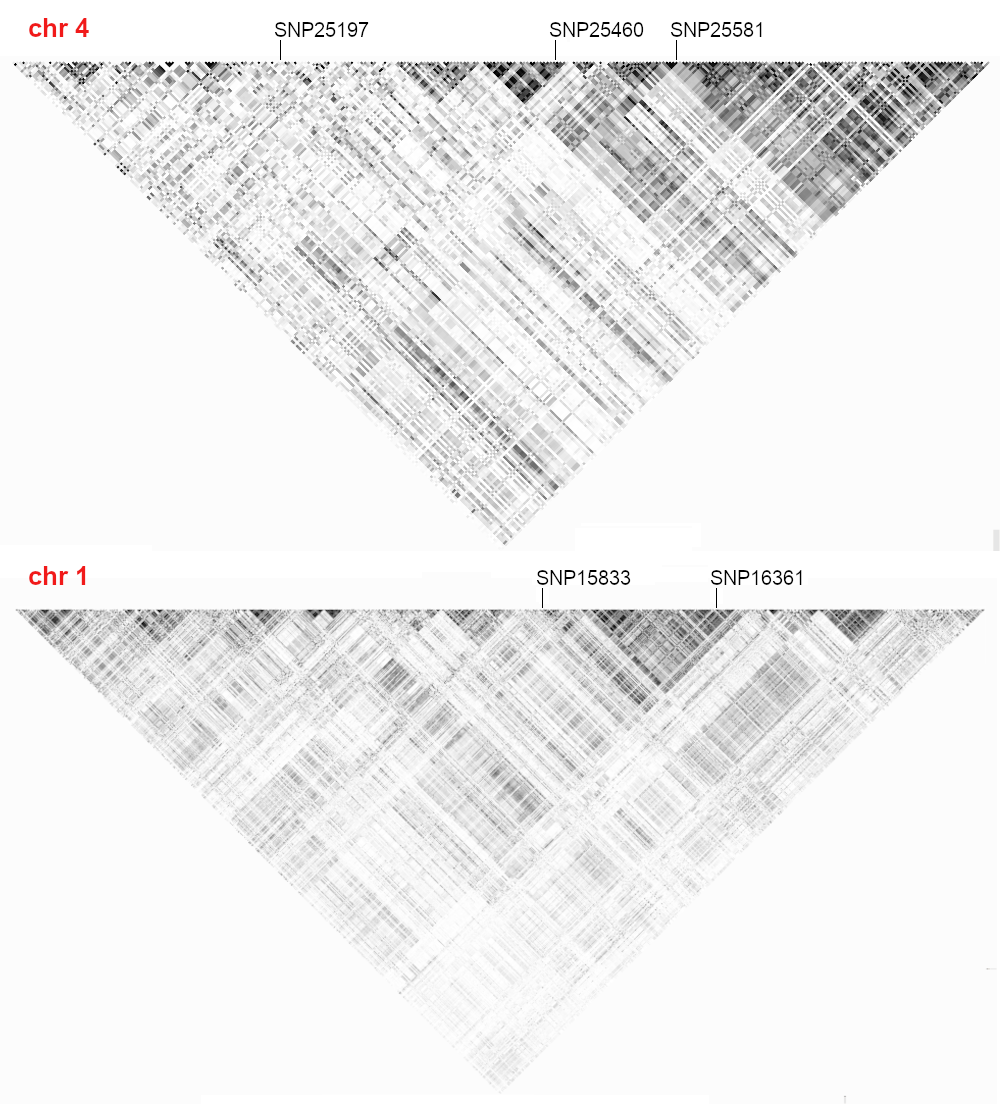
**

**Supplemental Figure 5**. Patterns of linkage disequilibrium around qMD1.1 and qMD4.1 loci.

**Supplemental Figure 6**

**
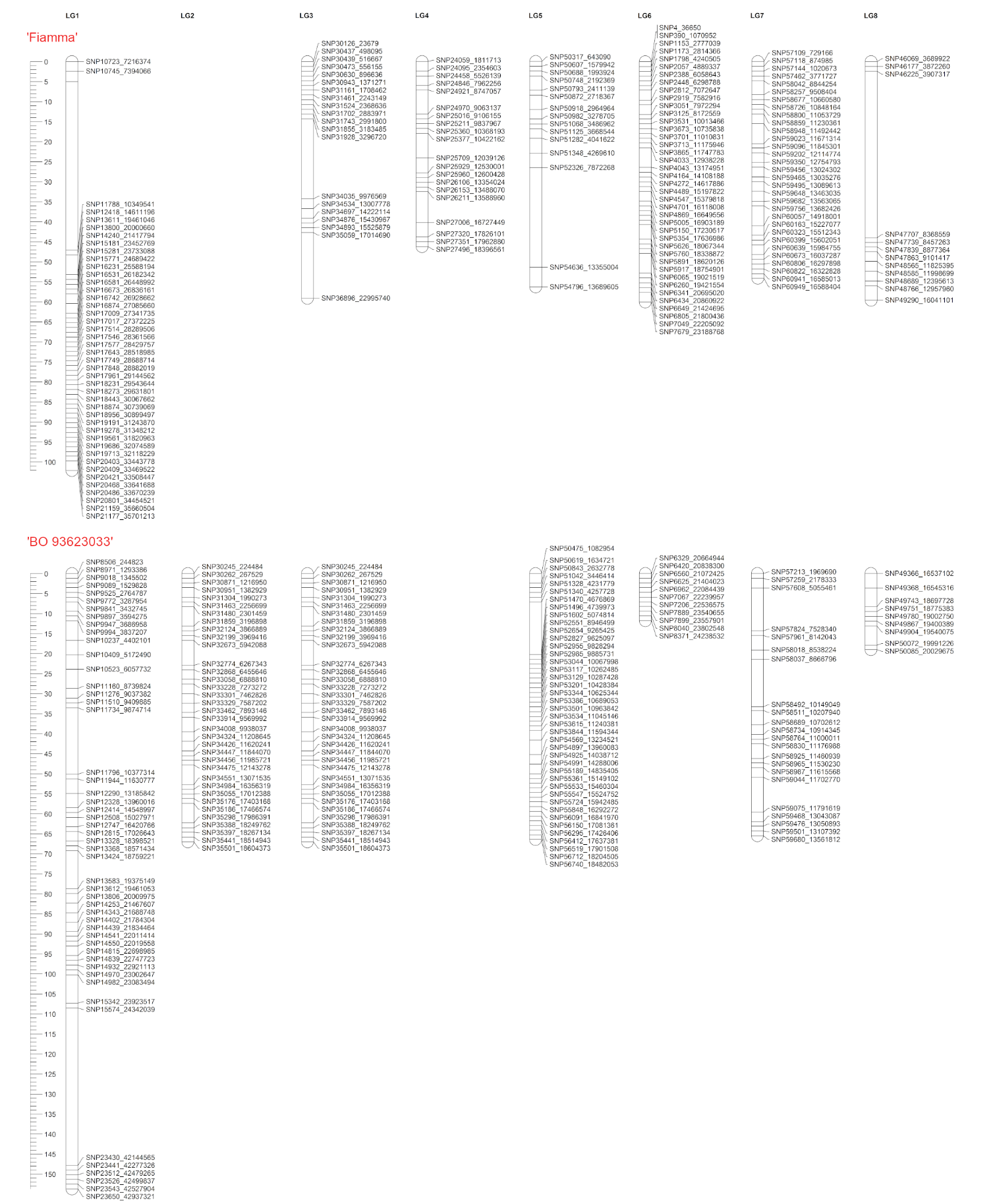
**

**Supplemental Figure 6**. Linkage map constructed from SPET-derived SNPs markers in the progeny derived from ‘Fiamma’ × ‘BO 93623033’ (F×B) F_1_ cross (n = 90 seedlings).

**Supplemental Figure 7**

**
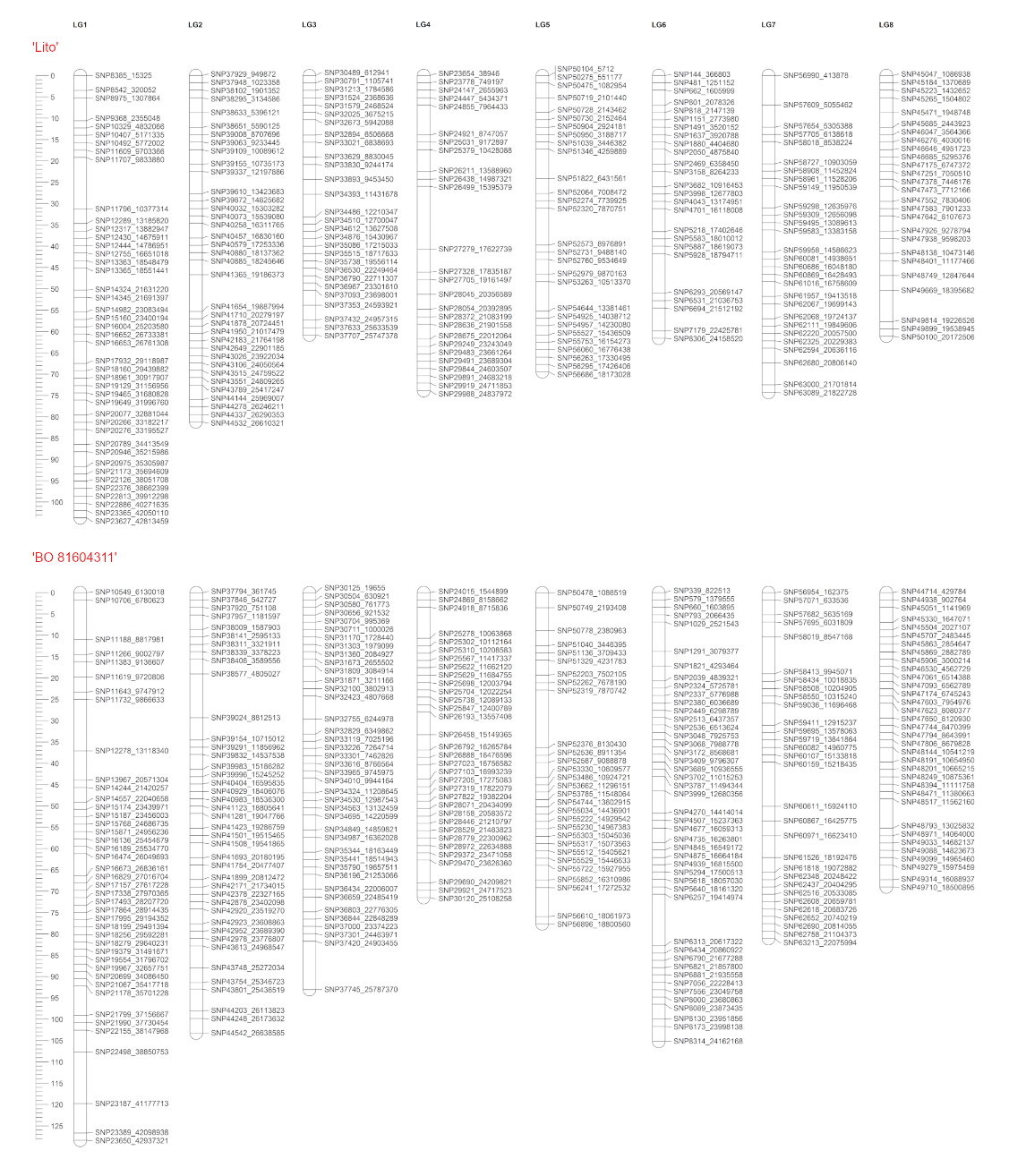
**

**Supplemental Figure 7**. Linkage map constructed from SPET-derived SNPs markers in the progeny derived from ‘Lito’ × ‘BO 81604311’ (L×B) F_1_ cross (n = 62 seedlings).
